# Modifications of the BAFF/BAFF-Receptor Axis in Patients With Pemphigus Treated With Rituximab Versus Standard Corticosteroid Regimen

**DOI:** 10.3389/fimmu.2021.666022

**Published:** 2021-05-14

**Authors:** Vivien Hébert, Maud Maho-Vaillant, Marie-Laure Golinski, Marie Petit, Gaëtan Riou, Olivier Boyer, Philippe Musette, Sébastien Calbo, Pascal Joly

**Affiliations:** ^1^ Normandie University, UNIROUEN, Inserm, U1234, FOCIS Center of Excellence PAn’THER, Rouen University Hospital, Department of Immunology and Biotherapy, Rouen, France; ^2^ Department of Dermatology, French Reference Center for Auto Immune Blistering Diseases, Rouen University Hospital, Normandie University, Rouen, France; ^3^ Paris Sorbonne North University INSERM UMR 1125 and Dermatology Department Avicenne University Hospital, Bobigny, France

**Keywords:** Pemphigus, BAFF - B-cell activating factor, BAFF-receptor, rituximab, Corticosteroïd

## Abstract

The efficacy of the B-cell-depleting agent rituximab has been reported in immune diseases but relapses are frequent, suggesting the need for repeated infusions. The B-cell activating factor (BAFF) is an important factor for B cell survival, class switch recombination and selection of autoreactive B cells, as well as maintaining long-lived plasma cells. It has been hypothesized that relapses after rituximab might be due to the increase of serum BAFF levels. From the Ritux3 trial, we showed that baseline serum BAFF levels were higher in pemphigus patients than in healthy donors (308 ± 13 pg/mL versus 252 ± 28 pg/mL, p=0.037) and in patients with early relapse compared who didn’t (368 ± 92 vs 297 ± 118 pg/mL, p=0.036). Rituximab and high doses of CS alone have different effects on the BAFF/BAFF-R axis. Rituximab led to an increase of BAFF levels associated to a decreased mRNA (Day 0: 12.3 ± 7.6 AU vs Month 36: 3.3 ± 4.3 AU, p=0.01) and mean fluorescence intensity of BAFF-R in non-autoreactive (Day 0: 3232 vs Month 36: 1527, mean difference: 1705, 95%CI: 624 to 2786; p=0.002) as well as on reappearing autoreactive DSG-specific B cells (Day 0: 3873 vs Month 36: 2688, mean difference: 1185, 95%CI: -380 to 2750; p=0.20). Starting high doses of corticosteroids allowed a transitory decrease of serum BAFF levels that re-increased after doses tapering whereas it did not modify BAFF-R expression in autoreactive and non-autoreactive B cells. Our results suggest that the activation of autoreactive B cells at the onset of pemphigus is likely to be related to the presence of high BAFF serum levels and that the decreased BAFF-R expression after rituximab might be responsible for the delayed generation of memory B cells, resulting in a rather long period of mild pemphigus activity after rituximab therapy. Conversely, the incomplete B cell depletion and persistent BAFF-R expression associated with high BAFF serum levels might explain the high number of relapses in patients treated with CS alone.

## Introduction

Pemphigus is, in most cases, mediated by anti-desmoglein (DSG) 1 and anti-DSG3 autoantibodies (1, 2). The B-cell activating factor (BAFF) is an important factor for B cell survival, class switch recombination, selection of autoreactive B cells and maintaining long-lived plasma cells (3, 4). The BAFF-receptor (BAFF-R), expressed on the majority of B cells, is the key receptor involved in promoting B cell survival (5). In mice, overexpression of BAFF results in the development of autoimmune manifestations (6). In human, a correlation between BAFF levels and disease severity has been shown in several auto-immune diseases (7–9), leading to belimumab (an anti-BAFF-R monoclonal antibody) approval in systemic lupus erythematosus (SLE) by the Food and Drug Administration. Anti-BAFF-R monoclonal antibodies (mAb) are currently being tested in pemphigus.

The Ritux3 trial showed that first-line treatment with rituximab (RTX), an anti-CD20 mAb, combined with a short-term regimen of corticosteroids (CS) was more effective and safer than a standard regimen of long-term CS (10). Despite a rather low (12 of 46) relapse rate in the RTX group compared to the CS group (20/44), most of relapses (9/12) occurred quite early in the RTX group during the first 12 months after the start of treatment, whereas relapses in the CS group occurred more regularly during patients’ follow-up (7 during the 1^st^ year and 13 during the 2^nd^ year after the start of treatment). A high relapse rate has been consistently reported in the literature in patients with pemphigus who received only one cycle of rituximab (11–14), as well as in other auto-immune diseases. It has been hypothesized that some relapses after rituximab might be due to the increase of serum BAFF levels, which could promote the recovery of auto-reactive B cells (4, 15).

We used samples collected from patients treated with RTX or oral CS alone in the Ritux 3 trial to longitudinally assess the BAFF/BAFF-R axis. For this, we studied: i) serum BAFF levels, ii) BAFF-R mRNA in bulked total B cells and one-cell sorted autoreactive B cells, and iii) BAFF-R phenotypic expression in both treatment groups.

## Methods

### Clinical Trial

Ninety newly-diagnosed pemphigus patients were randomly assigned to receive a standard regimen of CS versus RTX associated with a short-term regimen of CS. Patients in the RTX group were treated with the autoimmune regimen (two infusions of 1000 mg of RTX at Day 0 and Day 15) and a maintenance treatment corresponding to two infusions of RTX of 500 mg at Month 12 and Month 18. They also received an initial dose of prednisone of 0.5 to 1 mg/kg/day, depending on initial pemphigus severity (moderate versus severe), which was rapidly tapered over 3 to 6 months. Patients assigned to the standard oral CS group were given an initial dose of prednisone of 1 to 1.5 mg/kg/day, with a progressive tapering over 12 to 18 months, depending on initial pemphigus severity.

### Patients

Blood samples from RTX-treated patients were analyzed before (Day 0) and 36 months after the initial rituximab infusion (Month 36), after recovery of B lymphocytes. Blood samples from CS-treated patients were analyzed before (Day 0), 12 months (Month 12) and 24 months (Month 24) after the start of CS therapy in order to perform biological analyses in patients still receiving CS treatment. The pemphigus disease area index (PDAI) score was used to assess pemphigus severity.

We performed a sequential analysis of serum BAFF levels by enzyme-linked immunosorbent assay (ELISA) in 88 patients (45 treated with RTX, 43 treated with CS), and 37 healthy donors (HD). We analyzed the transcriptomic and cytometric profiles of BAFF-R expression in cell sorted autoreactive DSG+ B lymphocytes, and in DSG- whole B lymphocytes from 10 patients before and after treatment with RTX or CS, as well as in 7 HD.

### Desmoglein-Specific B-Cell Staining

In order to analyse DSG1 and DSG3 specific B cells, peripheral blood mononuclear cells (PBMCs) were extracted from venous blood using Ficoll-Hypaque (Lymphoprep™, Oslo, Norway). Then, B cells were isolated using Dynabeads Untouched Human B-cells kit (Invitrogen™, Carlsbad, USA) according to manufacturer’s instructions. Then, purified B cells were incubated for 30 minutes at 4°C with histidine-tagged recombinant DSG1 or DSG3 (30 ng/µl).

### Flow Cytometry Analysis

First, cells were incubated with Fc Blocking Reagent (eBioscience) prior to staining. For live cell analyses, dead cells were excluded by staining with LIVE/DEAD Fixable Aqua Dead Cell Stain (Life Technologies). Then, the phenotype of B cells was determined by flow cytometry with anti-human antibodies directed against IgG (BD Biosciences), CD19, CD27, IgM, BAFF-R and TACI (BD Biosciences). Anti-histidine coupled with phycoerythrin (R&D Systems) was used to identify DSG-specific B cells. Data were collected by FACS ARIA III (BD Biosciences) and analyzed with FlowJo software 10 (TreeStar).

### One-Cell Sorting and Pre-Amplification

DSG-specific single B cells were sorted by FACS ARIA III into 96-well plates containing 10 µL Platinum Taq polymerase and SuperScript III reverse transcriptase (Invitrogen), a mixture of Taqman primer-probes at 0.2×concentration specific for the transcripts of interest and CellsDirect qRT-PCR buffer (Invitrogen). Immediately following cell sorting, samples were centrifuged, incubated at 55°C for 10 minutes, and subjected to 20 cycles of Polymerase Chain Reaction (PCR) (50°C for 15 minutes then 95°C for 15 seconds for the reverse transcription, followed by 20 cycles of 95°C for 15 seconds and 60°C for 4 minutes for amplification). Subsequent pre-amplified single-cell cDNA was stored at −20°C until analysis.

### Real-Time Quantitative Polymerase Chain Reaction

After ¼ dilution in TE buffer, each cDNA sample was then separated into 48 separate reactions for further quantitative Polymerase Chain Reaction (qPCR) using the BioMark 48.48 dynamic array nanofluidic chip (Fluidigm, Inc.). Briefly, following hydraulic chip priming, 48 pre-amplified cDNA samples were mixed with a mild detergent loading solution to allow capillary flow, and the samples were added to a 48.48 nanofluidic chip (Fluidigm, Inc.) along with 38 individual Taqman primer-probe mixtures listed in along with 38 other individual Taqman primer-probe mixtures (Applied Biosystems) specific for individual transcripts of interest. The chip was then thermocycled through 40 cycles and fluorescence in the FAM channel was detected using a CCD camera placed above the chip, normalized by ROX (6-carboxy-X-rhodamine) intensity. One hundred CD19+ cells and no-cell wells were used as positive and negative controls respectively. To limit potentially biased measurement, cells with less than 2 expressed genes among the 5 control genes (HPRT1, B2M, GUSB, TUBB and GAPDH) were excluded from the analysis. Data were analyzed using Real Time PCR Analysis software with or without normalization of the Ct value for each gene using GAPDH as calibrator gene. We considered that the cell expressed the gene if the Ct value was < 40 and if the expression curve was a sigmoid. However, positive control wells containing 100 CD19+ cells showed detectable expression levels of all tested cytokine genes. The amount of RNA contained in one cell was therefore possibly too low. We analyzed qPCR results in frequency of cytokine gene expressing B cells between different groups.

### BAFF Enzyme-Linked Immunosorbent Assay

The concentration of BAFF in patients’ serum (42 RTX-treated and 26 CS-treated patients) was determined using ELISA: DuoSet kit from R&D Systems (Minneapolis, MN, USA), according to the manufacturer’s instructions. BAFF levels were measured at Days 0, 90, 180, 365, 730 and Month 36.

### Statistical Analysis

Prism software was used for statistical analysis. Fisher exact test was used to compare the frequencies, the T-test to compare patients before and after treatment and one-way Anova for multiple comparisons. A p value of less than 0.05 was considered significant for all analyses.

## Results

### BAFF Levels in Pemphigus Patients

**Figure 1:**
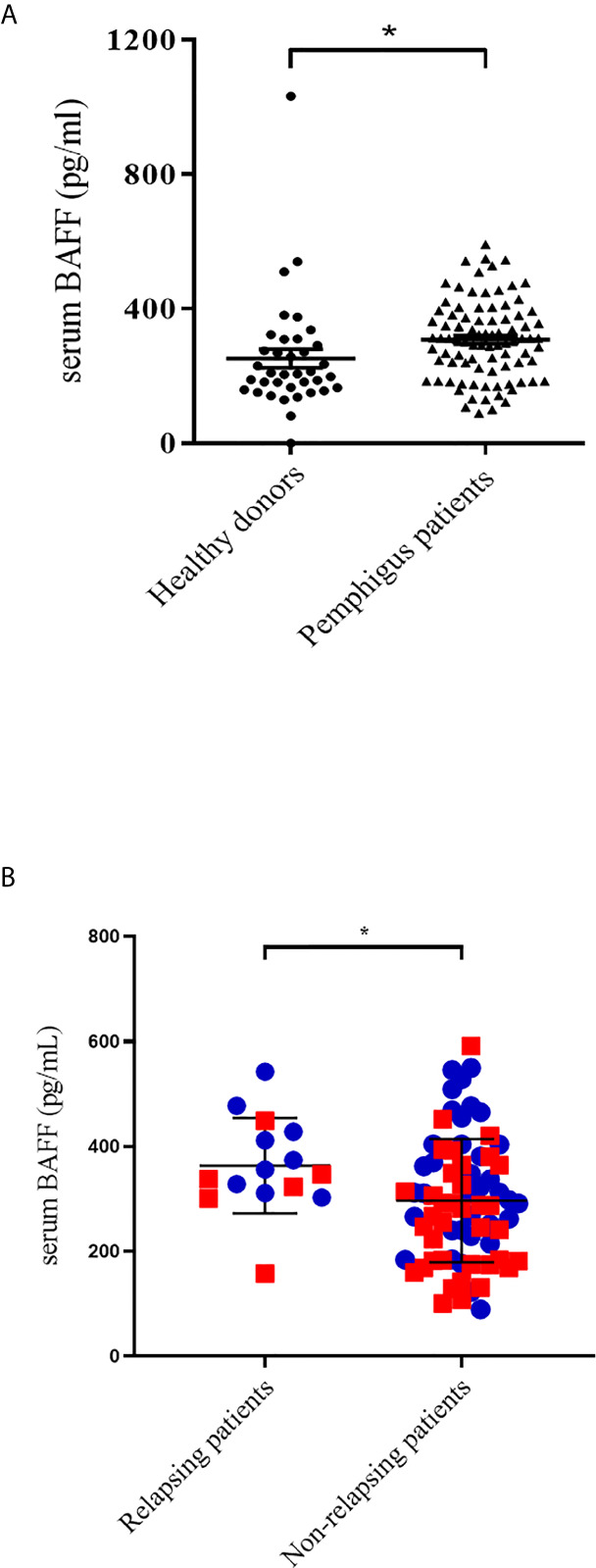
**(A)** Comparison of serum BAFF levels between healthy donors and pemphigus patients at baseline. **(B)** Comparison of baseline serum BAFF levels between short-term relapsing patients and non-relapsing patients. Blue circles represent rituximab-treated patients. Red squares represent corticosteroids-treated patients. *p < 0.05.

Using serum samples from 88 patients and 37 healthy donors (HD), we first showed that serum BAFF levels (mean ± SD) measured at baseline were significantly higher in pemphigus patients than in HD (308 ± 13 pg/mL vs 252 ± 28 pg/mL, p=0.037) (**Figure 1A**). Moreover, we showed that patients who further relapsed during the 12 months after the start of treatment had significantly higher serum BAFF levels than patients who did not relapse (368 ± 92 vs 297 ± 118 pg/mL, p=0.036) (**Figure 1B**). A ROC curve showed that a BAFF level ≥300 pg/mL provided a sensitivity of 54.8%, a specificity of 92.9%, and a 98% negative and a 28% positive predictive value for the occurrence of relapse during the first year after the start of treatment (AUC=0.70; 95% CI: 0.57-0.83; p=0.019), regardless of the treatment group (RTX or CS alone).

### Evolution of the BAFF/BAFF-R Axis After Rituximab

**Figure 2:**
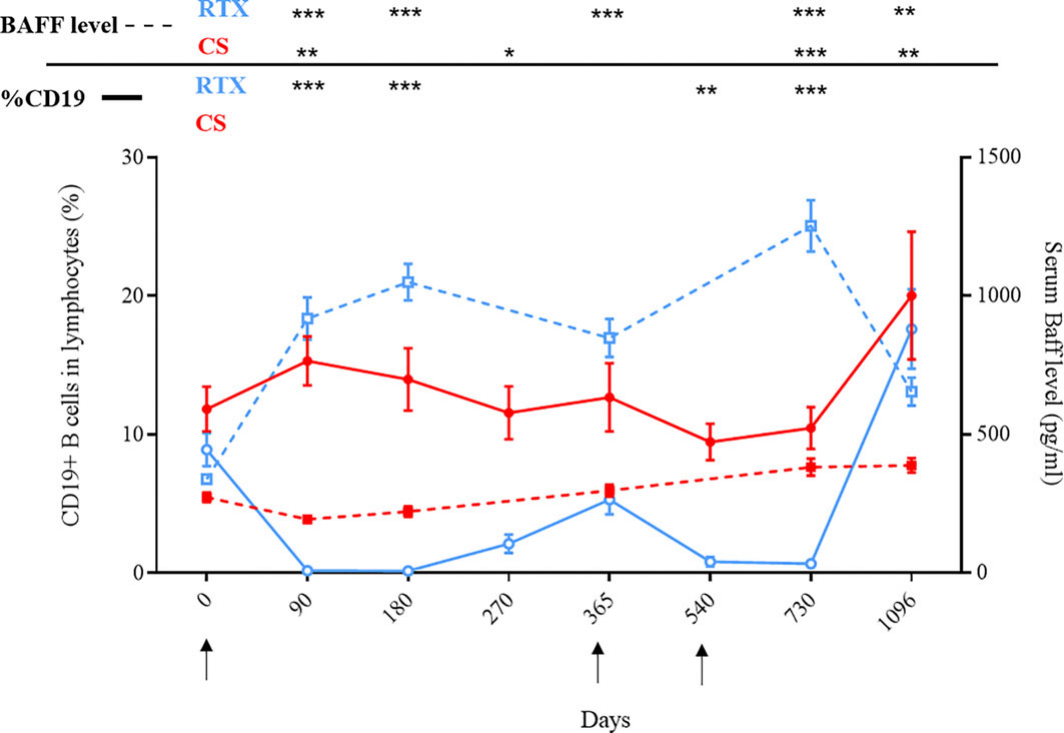
Evolution of serum BAFF levels and CD19+ B cells according to treatments: rituximab (blue curves) or corticosteroids (red curves). Mean percentages of CD19+ B cells among peripheral blood lymphocytes were measured by flow cytometry and are indicated in full lines. Mean serum BAFF levels were measured by ELISA and are indicated in dashed lines. Arrows correspond to rituximab infusions. *p < 0.05; **p < 0.01; ***p < 0.001.

Serum BAFF levels evolved inversely to the percentage of CD19+ blood B cells in patients from the RTX group. Serum BAFF levels started to decrease from Day 180 to Day 360, and from Day 730 to Day 1096, corresponding to periods of recovery of blood B cells (**Figure 2**).

**Figure 3:**
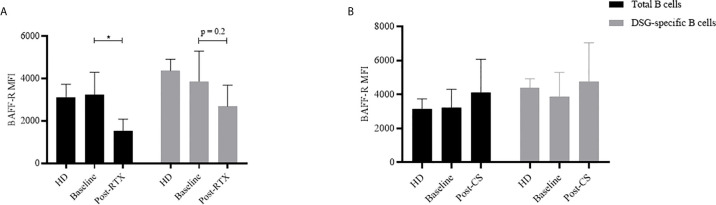
Evolution of B-cells BAFF-R expression according to treatment by rituximab **(A)** or corticosteroids **(B)**. **(A)** Evolution of the mean cytometric BAFF-R MFI expression in total B cells (black) and autoreactive DSG-specific B cells (grey) before (n = 22) and after rituximab (n = 11) and comparison to healthy donors (n = 4). **(B)** Evolution of the mean cytometric BAFF-R MFI expression in total B cells (black) and autoreactive DSG-specific B cells (grey) before (n = 22) and after corticosteroids (n = 11) and comparison to healthy donors (n = 4). *p < 0.05.

BAFF-R mRNA relative expression and BAFF-R protein expression were first studied in total B cells from 11 pemphigus patients. BAFF-R mRNA expression decreased after RTX treatment from 12.3 ± 7.6 AU at Day 0 to 3.3 ± 4.3 AU at Month 36, p=0.01, which corresponded to values close to those measured in HD (2.2 ± 2.1 AU) data not shown. The mean fluorescence intensity (MFI) of the BAFF-R protein in total B cells also decreased significantly after RTX from 3232 at Day 0 to 1527 at Month 36 (mean difference: 1705, 95% CI: 624 to 2786; p=0.002), the values measured at Month 36 were even lower than those measured in HD (**Figure 3A**).

We then assessed BAFF-R mRNA expression in one-cell sorted autoreactive B cells collected at baseline (n=191 cells) or after RTX (n=115 cells). MFI was measured in bulked autoreactive B cells from the same 11 patients. While we did not evidence significant modifications of BAFF-R mRNA expression after treatment (Day 0: 12/191 (6.3%) vs Month 36: 12/115, (10.5%); p =0.20), we observed a non-statistically significant decrease of BAFF-R phenotypic expression on reappearing autoreactive DSG-specific B cells relative to baseline samples (from 3873 at baseline to 2688 at Month 36, mean difference: 1185, 95% CI: -380 to 2750; p=0.20) (data not shown), which was reminiscent to that observed in the whole B cell population.

### Evolution of the BAFF/BAFF-R Axis After Corticosteroids Alone

Treatment with high doses of CS alone led to a transient but significant decrease of serum BAFF levels during the first three months after the start of treatment (273.2 pg/mL vs 194.2 pg/mL, p=0.0002), when patients received the highest doses of prednisone (**Figure 2**). Serum BAFF levels then re-increased from Day 90 to the end of the study, when prednisone doses were tapered under 20 mg/day.

mRNA and cytometric MFI expression of BAFF-R in total B cells did not show significant variations after CS treatment (mRNA expression: 2.2 ± 2.1 AU vs 10.1 ± 5.4; p=0.32; phenotypic expression: 3232 vs 4100, mean difference: 898, 95% CI: -2463 to 667; p=0.43). Longitudinal transcriptomic and cytometric analyses were then performed in one-cell sorted autoreactive B cells collected at baseline (n=191 cells) and after CS (n=120 cells), and in bulked autoreactive B cells, respectively. No significant modification of BAFF-R mRNA expression (12/191 (6.3%) vs 3/120, (2.5%); p=0.18), or phenotypic expression (3873 vs 4771, mean difference: -868, 95% CI: -2086 to 650; p=0.24) (**Figure 3B**) was evidenced between autoreactive cells collected at baseline and those collected after CS treatment.

## Discussion

In this study, we showed for the first time, in a significantly larger cohort than in previous studies, that pemphigus patients had higher BAFF serum levels than healthy donors (16, 17). Moreover, early-relapsing patients had higher baseline serum BAFF levels than patients who did not relapse during the first year of follow-up, regardless of the treatment used. Interestingly, we calculated that patients who had a baseline serum BAFF level <300 pg/mL had a 98% chance of remaining in clinical remission during the first year of treatment. Both groups of patients treated with RTX or corticosteroids who further short-term relapsed had a higher baseline mean serum BAFF level than patients who did not further relapse, although these differences did not reach statistical significance and may due to a weak number of relapses (data not shown).

We showed that RTX and high doses of CS alone had different effects on the BAFF/BAFF-R axis. As expected, the evolution of serum BAFF levels was negatively correlated with blood B cell count in the RTX group and serum BAFF levels started to decrease when B cells started to return. Interestingly, most relapses observed in the RTX group occurred precisely during that time when B cells were in presence of the highest BAFF serum levels (10, 18). On the contrary, CS alone led to a transient decrease of serum BAFF levels, followed by a re-increase after CS doses were tapered under 20 mg/day, corresponding to the time period during which most patients relapsed.

Our findings suggest that a combined therapy associating anti-CD20 and anti-BAFF monoclonal antibodies might be of interest in pemphigus. These biologics, which work through complementary mechanisms, might result in an enhanced depletion of circulating and tissue-resident autoreactive B lymphocytes when administered together. In particular, it would make sense to use anti-BAFF therapy when B cells start to return, when serum BAFF levels are still very high, i.e. around the sixth month after the initial RTX infusion. The therapeutic regimen currently proposed to prevent short-term relapses after the initial cycle of RTX in pemphigus, is to perform additional RTX infusions whose exact time interval after the initial cycle, dosage and number are not clearly determined. Another strategy to prevent these short-term relapses might be to combine RTX maintenance infusion and anti-BAFF therapy, or to use anti-BAFF therapy alone as maintenance therapy. A phase-3 study is currently conducted to evaluate and compare the efficacy and tolerance of subcutaneous injections of belimumab in association to 2 cycles of RTX or a placebo in patients with SLE, after promising results in refractory SLE (19, 20).

Second, we showed that RTX led to decreased mRNA and phenotypic expression of BAFF-R in non-autoreactive B cells. Such a modification has already been reported in total B cells from patients with rheumatoid arthritis and idiopathic thrombocytopenic purpura (21, 22). We also observed a decrease of BAFF-R phenotypic expression on reappearing autoreactive DSG-specific B cells, which likely did not reach statistical significance due to the limited number of sorted DSG-specific B cells. Nevertheless, one can hypothesize that the decreased BAFF-R expression after RTX might be responsible for the delayed generation of memory B cells, resulting in a rather long period of mild pemphigus activity after RTX therapy. Interestingly, these modifications of the BAFF/BAFF-R axis seem likely related to a specific effect of RTX, since we did not observe these modifications in patients treated with CS alone, in whom we did not observe any modification in BAFF-R expression.

The main strength of this work is the high number of sera in which BAFF dosages were longitudinally performed in particular in patients treated with RTX, which is currently the mainstay of treatment for moderate to severe types of pemphigus. Our principal limitation was the low number of autoreactive B cells that could be analyzed due to the rarity of this cell population in patients’ blood, and the quite long time after which some frozen samples were analyzed, which further lowered the number of auto-reactive cells that we were able to study. In particular, a higher number of sorted autoreactive B cells would have allowed us to reach a statistically significant difference, notably regarding the under-expression of the BAFF-receptor by recovering autoreactive B-cells.

Overall, these findings suggest that the activation of autoreactive B cells at the onset of pemphigus, is likely related to the presence of high BAFF serum levels. Furthermore, the decrease of BAFF-R expression observed in recovering B cells after RTX might prevent the binding of BAFF to these cells, resulting in a decreased activation of B cells despite the presence of high BAFF serum levels. Conversely, the incomplete B cell depletion and persistent BAFF-R expression associated with high BAFF serum levels might explain the high number of relapses in patients treated with CS alone.

## Data Availability Statement 

The original contributions presented in the study are included in the article. Further inquiries can be directed to the corresponding author.

## Ethics Statement

Ethical review and approval was not required for the study on human participants in accordance with the local legislation and institutional requirements. The patients/participants provided their written informed consent to participate in this study.

## Author Contributions

VH: Conceptualization, Investigation, Writing – original draft. MM-V: Investigation. M-LG: Investigation. MP: Investigation. GR: Software. OB: Resources. PM: Methodology. SC: Methodology. PJ: Writing – review & editing. All authors contributed to the article and approved the submitted version.

## Conflict of Interest

The authors declare that the research was conducted in the absence of any commercial or financial relationships that could be construed as a potential conflict of interest.
